# The Role and Dynamic of Strengthening in the Reconsolidation Process in a Human Declarative Memory: What Decides the Fate of Recent and Older Memories?

**DOI:** 10.1371/journal.pone.0061688

**Published:** 2013-04-26

**Authors:** Cecilia Forcato, Rodrigo S. Fernandez, María E. Pedreira

**Affiliations:** Laboratorio de Neurobiología de la Memoria, Departamento de Fisiología, Biología Molecular y Celular, Facultad de Ciencias Exactas y Naturales, Universidad de Buenos Aires, IFIBYNE – CONICET, Buenos Aires, Argentina; Peking University, China

## Abstract

Several reports have shown that after specific reminders are presented, consolidated memories pass from a stable state to one in which the memory is reactivated. This reactivation implies that memories are labile and susceptible to amnesic agents. This susceptibility decreases over time and leads to a re-stabilization phase usually known as reconsolidation. With respect to the biological role of reconsolidation, two functions have been proposed. First, the reconsolidation process allows new information to be integrated into the background of the original memory; second, it strengthens the original memory. We have previously demonstrated that both of these functions occur in the reconsolidation of human declarative memories. Our paradigm consisted of learning verbal material (lists of five pairs of nonsense syllables) acquired by a training process (L1-training) on Day 1 of our experiment. After this declarative memory is consolidated, it can be made labile by presenting a specific reminder. After this, the memory passes through a subsequent stabilization process. Strengthening creates a new scenario for the reconsolidation process; this function represents a new factor that may transform the dynamic of memories. First, we analyzed whether the repeated labilization-reconsolidation processes maintained the memory for longer periods of time. We showed that at least one labilization-reconsolidation process strengthens a memory via evaluation 5 days after its re-stabilization. We also demonstrated that this effect is not triggered by retrieval only. We then analyzed the way strengthening modified the effect of an amnesic agent that was presented immediately after repeated labilizations. The repeated labilization-reconsolidation processes made the memory more resistant to interference during re-stabilization. Finally, we evaluated whether the effect of strengthening may depend on the age of the memory. We found that the effect of strengthening did depend on the age of the memory. Forgetting may represent a process that weakens the effect of strengthening.

## Introduction

Animals’ brains constantly encode the features of their surrounding environment; this is a critical function for everyday animal survival as well as for learning new information to successfully interact with the external world. In this context, the process of transforming new information into long-lasting memory has been of great interest in neurobiology over the last century. The seminal studies of Müller and Pilzecker [1] using verbal learning led to the idea that memories become enduring through a process of consolidation. This theory assumes that memories are labile during a window of time after acquisition; however, memories become stable and resistant to amnesic agents over time. It is assumed that consolidation is a conserved evolutionary process that requires an initial phase of RNA and protein synthesis [2], [3]. However, the notion that memories are immutable after consolidation has been changed. Since the early study of Misanin et al. [4], several reports have shown that after the presentation of a specific reminder, old memories pass from a stable state to reactivated one. Reactivation implies that the memory is labile and once again susceptible to amnesic agents. This susceptibility decreases over time leading to a re-stabilization phase, which is usually known as reconsolidation. Reconsolidation may share many of the cellular and molecular mechanisms used during consolidation [5], [6]. However, the term reconsolidation does not represent the recapitulation of initial consolidation, but rather, it refers to the functional role of this process: to stabilize memories [7].

Regarding the biological role of the labilization-reconsolidation process, two functions have been proposed [7], [8]. One hypothesis suggests that the destabilization of the original memory that occurs after a reminder allows new information into the background of the original memory to be integrated (memory updating; [9], [10]. The other hypothesis suggests that the labilization-reconsolidation process strengthens the original memory (memory strengthening; [11], [12]). With respect to strengthening, Lee [11] found that a second learning trial enhanced (or strengthened) a contextual fear memory that had been consolidated; however, this occurred only after destabilization. In this report and given that reconsolidation may be isolated from initial memory consolidation using doubly dissociable mechanisms of hippocampal contextual fear memories [13] Lee demonstrated doubly dissociable hippocampal mechanisms occurring in the first and second learning trials. In another report using a rat inhibitory avoidance paradigm, Inda et al. [12] tested whether reconsolidation mediates memory strengthening and examined its interaction over time. They found that successive reactivations of recent memories by re-exposition to the context of the original memory resulted in reconsolidation that mediated memory strengthening and prevented forgetting; this effect was temporally limited.

We previously demonstrated that reconsolidation of human declarative memories serves both functions [14], [15]. Our paradigm consists of a verbal learning task (lists of five pairs of nonsense syllables) using a training process (L1-training) on Day 1 of the experiment. After the declarative memory is consolidated, it can be labilized via presentation of a specific reminder. The memory then passes through a stabilization process. To reveal the presence of this process, we used a second learning task (L2-training), which interfered with the re-stabilization phase of the original memory. Furthermore, the labilization-reconsolidation was only triggered under certain circumstances. When the reminder was formed by the context cues and one cue syllable, without giving the subjects the opportunity to write down the response syllable (cue-reminder), the labilization-reconsolidation was triggered. In contrast, when the reminder only included contextual cues (context reminder), the memory was evoked but not labilized. Thus, as in other paradigms, the presence of a mismatch component, a discrepancy between expected and current events in the reminder, determined whether reconsolidation occurred [16], [17].

To examine strengthening attributed to reconsolidation, in a previous report [15] we triggered labilization-reconsolidation processes successively by repeated presentations of the proper reminder (cue-reminder). The memory was enhanced when at least a second reminder was presented within the time window of the first labilization-reconsolidation process induced by the first reminder. However, improvement was revealed only when at least two reminders were presented; additionally, it was not a consequence of retrieval only. That is to say, the contextual-cues only evoke the memory, but the memory remains stable.

Demonstrating strengthening creates a new scenario for the reconsolidation process; strengthening is thus a new factor that may transform the dynamics of memory. Therefore, new questions emerge, which are based on the fact that improvement may compromise the fate of a memory. The goal of this research was to evaluate the role of strengthening in the reconsolidation process, using a declarative memory paradigm in humans under various conditions.

First, we analyzed whether strengthening of the original memory by repeated labilization-reconsolidations maintained the memory available for longer periods of time [12]. We investigated whether strengthening not only increases the precision of the memory [15] but also augments its persistence. Strengthening was demonstrated during acquisition; this process made interfering agents after labilization less effective [18]. Considering this, we analyzed how strengthening via repeated labilization-reconsolidation processes modified the effect of an amnesic agent presented immediately after subsequent labilizations. Finally, considering that older memories are resistant to reactivation [6], [19] we evaluated whether the effect of strengthening could depend on the age of the memory.

We found that just one labilization-reconsolidation process was enough to strengthen a memory that was evaluated 5 days following its re-stabilization. We also demonstrated that this effect was not triggered by retrieval only. Our results indicated that repeated labilization-reconsolidations rendered memories more resistant to interference during the re-stabilization phase. Finally, strengthening appeared to depend on the age of the memory. In this case, forgetting could be considered a process that weakens the effect of this function. Overall, considering that this study examined strengthening in various experimental scenarios, this report may shed light onto the role of reconsolidation in the fate of declarative memories in humans.

## Results

### Memory Persistence is Increased by Repeated Triggering of Labilization-reconsolidation

**Figure 1:**
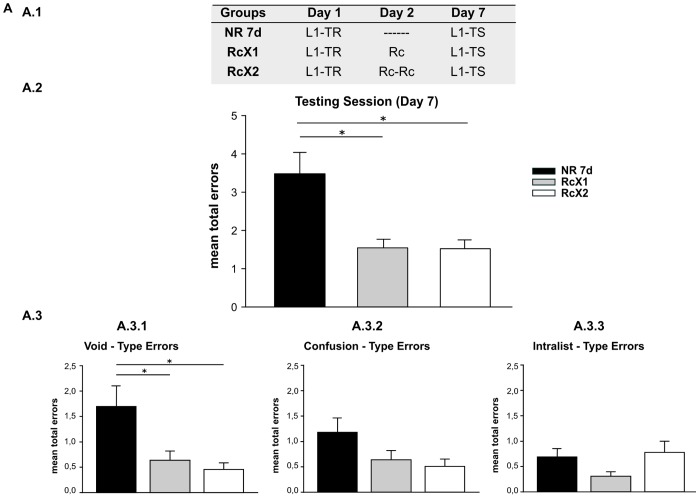
Memory strengthening by repeated triggering of labilization-reconsolidation modified the memory persistence. **A) Experiment 1.A (n = 12). A.1) Experimental protocol.** A three-day experiment. L1-TR, stands for the training session of the list of syllables (List 1; **L1**), Rc for the cue reminder, and L1-TS for the testing session of L1. Groups differ in the number or presence or absence of reminders that they received on Day 2. Group NR 7d without any reminder presentation, Group RcX1 received a cue reminder, Group RcX2 received two cue reminders. **A.2) Testing Session.** Mean number of total errors +/− SEM on Day 7. *,p<0,05. Black bar stands for Group NR 7d, Light Gray bar for Group RcX1 and White bar for Group RcX2. **A.3) Error Type. A.3.1)** Mean number of Void-Type error errors +/− SEM on Day 7. **A.3.2)** Confusion-type errors. **A.3.3)** Intralist-type errors. Symbols as above.

To evaluate how memories were strengthened by repeated reactivations and how this strengthening modifies memory persistence, we conducted a seven-day experiment using three groups (Figure 1A.1). On Day 1, subjects learned a list of five pairs of cue-response syllables (training session). On Day 2, two groups received a treatment. In this experiment, the treatment was the presentation of varying numbers of cue-reminders. We have previously demonstrated that this type of reminder triggers the labilization-reconsolidation process [20]. Thus, the one cue-reminder group received one cue-reminder (RcX1) and the two cue-reminder group (Rcx2) received two cue-reminders, which were separated by a five-min interval. The cue-reminder was formed by the specific context associated with the list of syllables plus one cue-syllable. Participants were not given an opportunity to write response syllables. Finally, the non-reminder group did not receive any treatment on Day 2 (NR 7d). All subjects underwent testing on Day 7. We also categorized the types of errors participants made during testing [15].

#### One or two cue-reminders improve performance on Day 7

A repeated-measures ANOVA revealed no significant differences between the groups at training (Figure S3.A, F(2,33) = 1,414, p = 0,257) as well as no group by trial interaction (F(16,264) = 0,735; p = 0,756). Moreover, an analysis of the percentage of correct responses given during the last four training trials yielded no significant difference between groups during the training session, (Figure S3.A inset, F(2,33) = 0,802, p = 0,456).

The performance of each group on Day 7 was estimated using the mean of total errors made when responding to the cue-syllables during the two testing trials. The subjects who received one or two cue reminders on Day 2 performed better than those who did not receive any reminders (Figure 1A.2). Specifically, the Rcx1 and Rcx2 groups made fewer errors than the NR group during the two testing trials (One-way ANOVA F(2,33) = 7,405, p = 0,002; LSD post-hoc Comparison p = 0,001, p = 0,002, respectively). We then analyzed the types of errors made during testing and classified them into three categories. The difference between the types of errors made by each group was notable (Figure 1A.3). Confusion-type errors (writing a non-existent response syllable) and the intra-list type (writing response syllables for a different cue syllable) were similar between the three groups (Figure 1A.3.2; F(2,33) = 2,054, p = 0,144; F(2,33) = 1,026, p = 0,369, respectively). However, the RcX1 and Rcx2 groups made fewer void-type errors (blank responses) than did the NR group. Indeed, significant differences were revealed between groups upon testing (Figure 1A.3.1, One-way ANOVA F(2,33) = 5,878 p = 0,006; LSD post-hoc comparison p = 0,007 p = 0,004).

This first result suggests that the memory improvement that was triggered by just one or two consecutive labilization-reconsolidations could be observed on Day 7. This supports the idea that the memory is available for longer periods of time, as was similarly demonstrated in rat models of aversive memories [12].

We have shown that omitting one aspect of a reminder retrieves the memory but deactivates the labilization-reconsolidation process. More specifically, it is possible to recover the stored information, but this information is protected from modifications because the memory trace is still stable. Indeed, the presentation of the context alone (music, light and image) evokes the target memory. Instead, the inclusion of one cue syllable in the context without the possibility to answer triggers the reconsolidation process. Thus, our paradigm offers various reminders [20] to distinguish between these contrasting interpretations, namely, that memory retrieval, rather than memory reactivation. The memory reactivation is the unique condition associated with the reconsolidation process and the previous mandatory step before the re-stabilization [16]. In a recent report, we demonstrated that repeated destabilization of the original memory can strengthen it if subsequent destabilizations occur in the time window of the first re-stabilization. This effect depends on successively triggering reconsolidation, not from successive retrievals [15]. Consequently, here, to refute the notion that this effect could be due simply to retrieval, we evaluated the effect of retrieval only on strengthening the target memory and consequently changing its persistence, using a context reminder which only evoked the target memory (Text S1 and Figure S1). Subjects who received one cue reminder made fewer errors than subjects who were given one context reminder on Day 2 during the two testing trials on Day 7. Therefore, retrieval on Day 2 did not increase memory persistence; this effect depends on the occurrence of at least the presentation of one cue-reminder to labilize the declarative memory. It is important to stress that a clear difference appear when one cue reminder is presented and the testing session occurs 24 or 120 h later. Under the last condition (Figure 1A.2) one cue-reminder strengthens the target memory. However, this effect has not been observed when the evaluation occurs 24 h after the reactivation [15] The difference in the intersession interval (between the reactivation and the testing session) might affect the possibility to reveal the same effect. A ceiling effect (when we considered the number of errors committed at testing) might overshadow the one cue reminder strengthening-effect.

### Memories Strengthened by Repeated Labilization-reconsolidations are more Resistant to Interference of a Second Task

As mentioned above, we reported that a human declarative memory undergoes reconsolidation [21]. To common way to reveal the presence of such process, is to present an amnesic agent after the reactivation to interfere the re-stabilization of the memory trace. Thus, the presence of the reconsolidation process is revealed by the absence (impairment) of the memory at testing session [16]. In our case, the tool selected to demonstrate reconsolidation was a second learning task; this task served as an interfering agent to impair re-stabilization of a reactivated memory. For this paradigm, we proposed an alternative method to reveal the amnesic effect of interfering agents on the target memory. It takes into account the fact that memories are integrated into complex associative networks, and accordingly, the activation of one memory may interfere with the desired retrieval. This method is based on a temporal “forgetting” effect. This effect states that retrieval of target memories could temporarily block subsequent retrieval of other, related memories; this is termed retrieval-induced forgetting (RIF) [22], [23].

When a target memory is intact, its retrieval may interfere with subsequent retrievals of related memories (RIF). As a consequence, a poor performance was expected for the second task at testing. Otherwise, when a target memory is impaired, its retrieval will not interfere with the retrieval of related memories (No-RIF [21]). Accordingly, a high performance was predicted at testing.

Using this framework, we designed a three-day experiment combining repeated reactivations immediately following a new learning task. Here, our working hypothesis stated that No-RIF effect on List 2 could be detected because the reconsolidation of the target memory was impaired by the second learning. Hence, we evaluated impairment of the target memory first by analyzing the presence of RIF on List 2 and then by comparing performance between groups on List 1.

**Figure 2:**
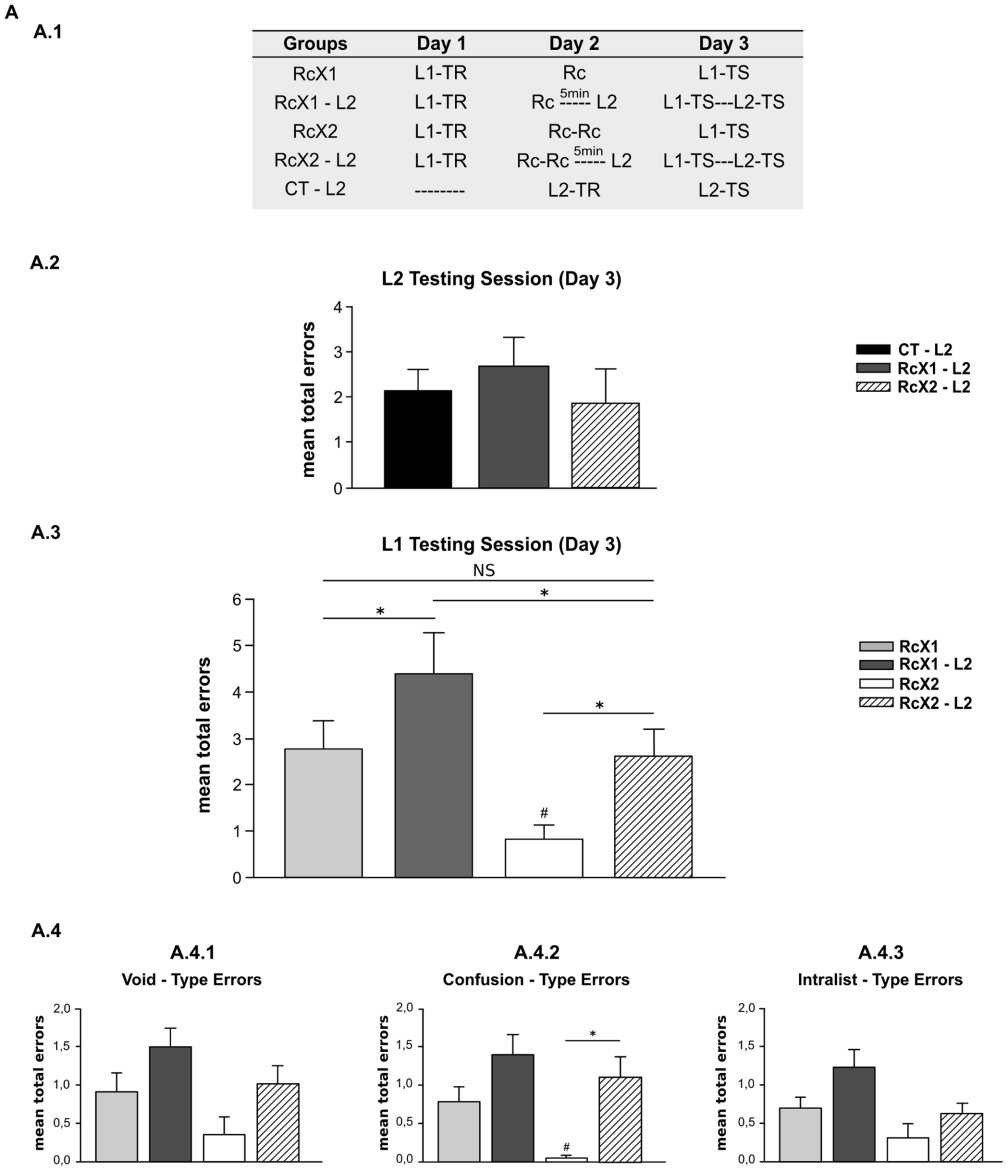
Strengthened memories by repeated labilization-reconsolidations are more resistant to be interfered with a second task. **A) Experiment 2.A (n = 13). A.1) Experimental protocol.** A three-day experiment**.** Rc–L2, stands for the presentation of the cue reminder and five minutes later the acquisition of the Interference task List-2 (**L2**), L2-TR for the training in L2, L2-TS for the testing of that list and the remaining symbols as in Experiment 1. Groups differ in the number of reminders received and the presence or absence of the L2 list. Groups RcX1 and RcX2 as in Experiment 1. Group RcX1– L2 received a cue reminder and five minutes later learned L2 list, Group RcX2– L2 received two cue reminder and five minutes later learned L2 list, Group CT-L2 only learned L2 list on Day 2. **A.2) L2 Testing Session.** Mean number of total errors +/− SEM on Day 3. Black bar stands for Group CT-L2, Dark Gray bar for Group RcX1-L2 and Stripe bar for Group RcX2-L2. **A.3) L1 Testing Session.** Mean number of total errors +/− SEM on Day 3. *, p<0,05. Symbols as in Experiment A.2 and Experiment 2 A.2. **A.4) Error Type. A.4.1)** Mean number of Void-Type error errors +/− SEM on Day 7. **A.4.2)** Confusion-type errors. **A.4.3)** Intralist-type errors. Symbols as above.

The experiment included five groups (Figure 2A.1). On Day 1, the subjects of four of these groups received a training session (List 1). On Day 2, they received a treatment (one or two presentations of the cue reminder); some groups received the second learning task (List 2), which served as an amnesic agent. The remaining group learned List 2 only. Thus, the one cue-reminder group received one cue reminder (Rcx1); the interfering cue-reminder group received a cue reminder and immediately learned List 2 (RcX1-L2); the two cue-reminder group (Rcx2) received two cue reminders separated by a five-min interval; the interfering two cue-reminders group received two cue reminders and immediately learned List 2 (RcX2-L2); and finally, the list 2 group, learned List 2 (L2). All subjects received the testing session on Day 3. The groups that learned both lists were tested first for the target memory (List 1); after a 5-min delay, they were tested for the interfering memory (List 2). The L2 group was evaluated for List 2 only. As before, we analyzed the types of errors made during testing [15]; the analysis was centered on the fact that the variation in error types may reflect different effects.

#### The absence of RIF in List-2 testing indicates an interfering effect on the reactivated memory

A repeated-measures ANOVA revealed no differences between the groups at List 2 training (Figure S3.C, F(2,36) = 2,155, p = 0,130) as well as no group by trial interaction (F(16,288) = 0,938, p = 0,525). Moreover, an analysis of the percentage of correct responses given during the last four training trials yielded no significant differences between groups at training (Figure S3.C inset, F(2,36) = 1,828, p = 0,175).

The performance for List 2 on Day 3 in each group was estimated using the mean of total errors made when responding to List 2 cue syllables during testing. Subjects who received one or two cue reminders on Day 2 and List 2 directly after behaved similarly to the group that learned List 2 on Day 2 only (FIG. 2A.2). Thus, for List 2, the RcX1-L2 RcX2-L2 and L2 groups made a similar number of errors F(2,36) = 0,757, p = 0,475). This result revealed the absence of RIF; according to our previous results, re-stabilization of the List 1 memory was impaired in these groups. We subsequently analyzed List 1 performance considering the number of errors made on List 1 as an indicator of the amnesic effect induced by the interfering agent [20], [21].

#### Two cue reminders given prior to the interference task also improves performance of target memory on Day 3

A repeated-measures ANOVA revealed no significant differences between the groups at List 1 training (Figure S3.D, F(3,48) = 0,641, p = 0,592) as well as no group by trial interaction (F(24,384) = 0,880, p = 0,629). Moreover, an analysis of the percentage of correct responses given during the last four training trials yielded no significant difference between groups at training (Figure S3.D inset, F(3,48) = 0,711, p = 0,549).

The List 1 performance on Day 3 for each group was estimated using the mean of the total errors made when responding to the cue-syllables of the two testing trials. Subjects who received two successive cue reminders on Day 2 performed better than those who received only one reminder (RcX1 Figure 2A.3). Specifically, the Rcx2 group made fewer errors than the RcX1 group during the two testing trials (One-way ANOVA F(3,48) = 7,927, p = 0,0002; LSD post-hoc Comparison p = 0,017). Regarding error type, the Rcx2 group made fewer confusion-type errors (writing non-existent response syllables) than the RCX1 group (Figure 2A.4.2, One-way ANOVA F (3,45) = 4,542 p = ;0,007 LSD post-hoc comparison p = 0,038). This result confirmed that successively triggering at least two labilization-reconsolidations improved retention of consolidated declarative memories [15]. Considering the effect of interference, we found effects that were expected when comparing interference groups with their respective control group. Thus, the subjects who received one cue-reminder followed by the List 2 learning task (RCX1-L2), showed a greater number of total errors than the subjects who received one cue reminder only (RCX1) (One Way ANOVA F(3,48) = 7,297, p = 0,0002 LSD post-hoc comparison p = 0,019). Furthermore, the same results profile was obtained by the group that received 2 cue reminders followed by the List 2 learning task (RcX2-L2) compared its respective control group, who received 2 cue reminders only (RcX2) (p = 0,0182). More interestingly, similar performance was observed in the group that received two cue reminders immediately following interference (RcX2-L2) and the group that receive one reminder only (Rcx1). No significant differences were observed between these groups (p = 0,981). We also found a significant difference between the groups that received the interfering agent (List 2; RcX1-L2 and RcX2-L2) p = 0,0187.

The error type analysis reflected effects described by the total number of errors. Thus, fewer confusion-type errors (writing non-existent response syllables) were observed in the group that received two successive cue reminders (RcX2) compared to the group that received only one cue reminder (RcX1); this indicated that strengthening occurred (Figure 2A.4.2 One-way ANOVA F(3,45) = 4,542, p = 0,007). This result also confirmed the relationship between strengthening and improvement in memory precision. Additionally, other differences emerged in this experiment. The comparison between the interference groups and their respective controls showed an increase in the amount of confusion-type errors in the groups that learned the second task after two cue reminders (RcX2 and RcX2L2 p = 0,012). It also revealed the absence of differences between the RcX2L2 and RCX1 groups, as was shown by the total number of errors (p = 0,981).

As a whole, these results introduce the possibility that two processes may coexist that are dependent on labilization of target memories. These processes include strengthening the memory via repeated reactivations, and interference of the second learning task on re-stabilizations of the target memory. As a consequence, interference is less effective: the memory is preserved in some way by enhancement produced by successive labilization-reconsolidations. Finally, these results replicated previous one, being only two cue reminders the effective treatment to strength the target memory.

### Successive Labilization-reconsolidation Processes do not Strengthen older Declarative Memories on Day 8

Up to this point, the analysis of strengthening was performed using one type of experimental protocol; whereas the treatment to induce memory improvement was performed 24 h after memory acquisition, when the memory had been consolidated [21]. Although, it is also well known that in some paradigms, the length of the interval between memory acquisition and memory reactivation may compromise destabilization of the target memory [6], [7].

In this scenario, our working hypothesis stated that strengthening via repeated labilization-reconsolidation processes may depend on the age of the memory. We studied whether strengthening improved retention of a declarative memory that was reactivated 7 days after acquisition.

**Figure 3:**
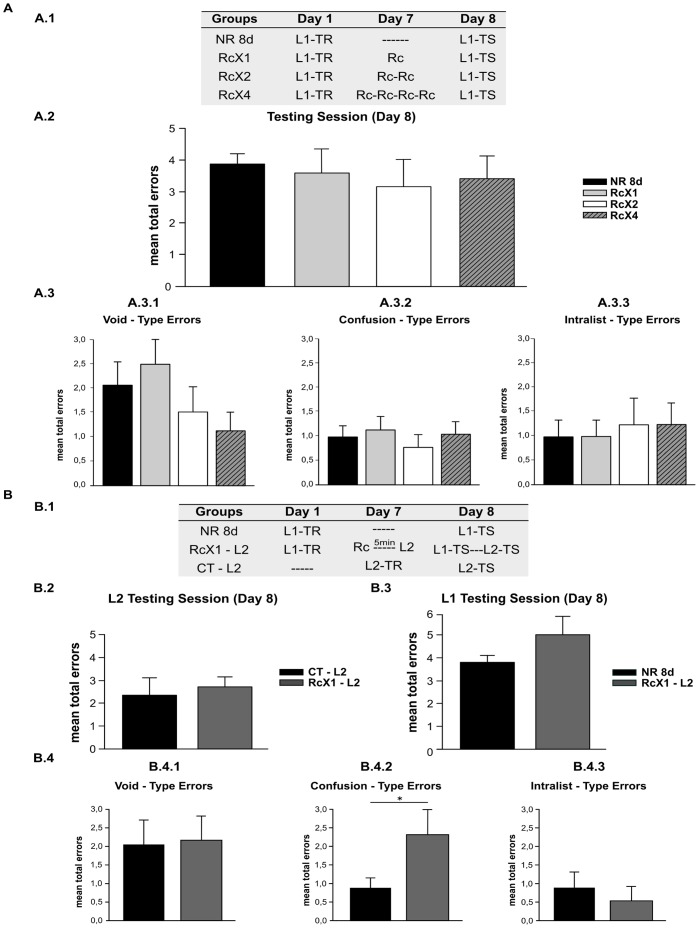
Successive labilization-reconsolidation processes do not strengthen older declarative memory. **A)** Two or four cue-reminders do not improve performance on Day 8. **Experiment 3.A (n = 10). A.1) Experimental protocol.** A three-day experiment. Symbols as in Experiment 1.A. Groups differ in the number or presence of reminders that they received on Day 7. Group NR 8d received no reminder, RcX4 received the cue reminder four times, Groups RcX1 and RcX2 as in Experiment 1.A. **A.2) Testing Session.** Mean number of total errors +/− SEM on Day 8. Black bar stands for Group NR 8d, Light Gray bar for Group RcX1, White bar for RcX2, and Stripe gray bar for Group RcX4. **A.3) Error Type. A.3.1)** Mean number of Void-Type error errors +/− SEM on Day 7. **A.3.2)** Confusion-type errors. **A.3.3)** Intralist-type errors. Symbols as above. **B) Older memories are subject to change by forgetting compromising the effect of the interference. Experiment 3B (n = 10). B.1) Experimental protocol.** A three-day experiment. Symbols as above. **B.2) L2 Testing Session.** Mean number of total errors +/− SEM on Day 8. Black bar stands for Group CT-L2, Dark Gray bar for Group RcX1-L2. **B.3) L1 Testing Session.** Mean number of total errors +/− SEM on Day 8, Black bar stands for NR 8d and Dark gray for RcX1-L2. **B.4) Error Type. B.4.1)** Mean number of Void-Type error errors +/− SEM *, p<0,05. on Day 8. **B.4.2)** Confusion-type errors. **B.4.3)** Intralist-type errors. Symbols as above.

To determine the effect of repeatedly triggering labilization-reconsolidation on the strengthening of older target memories, we conducted an eight-day experiment using four groups (Figure 3A.1). On Day 1, subjects learned a list of paired syllables (List 1). On Day 7, three groups received a treatment. The one-cue reminder group received one cue reminder (RcX1); the two-cue reminder group received two cue reminders (Rcx2); and the four-cue reminder group received 4 cue reminders that were separated by five min between each (RcX4). Finally, the NR group did not receive any treatment on Day 7. All subjects were tested on Day 8.

#### Two or four cue-reminders do not improve performance on Day 8

A repeated-measures ANOVA revealed no significant differences between the groups at training (Figure S3.E, F(3,36) = 0,305, p = 0,820) as well as no group by trial interaction (F(24,288) = 1,325, p = 0,145). Moreover, an analysis of the percentage of correct responses given during the last four training trials yielded no significant difference between the groups at training (Figure S3.E inset, F(3,36) = 0,206, p = 0,995).

An analysis of the performance of each group on Day 8 revealed that the subjects who received two or four successive cue reminders on Day 7 performed similarly to those who received one or none (Figure 3A.2). Specifically, the RcX1, Rcx2, Rcx4 and NR 7d groups made a similar number of errors during the two testing trials (One-way ANOVA F(3,36) = 0,302, p = 0,823). Similar results were obtained for the analysis of the error types; a similar number of errors each error type was observed between groups (Figure 3A.3; Confusion type errors (F(3,36) = 0,398 p = 0,755); intra-list type errors F(3,36) = 0,127 p = 0,943; Void type errors F(3,36) = 1,476 p = 0,237).

These results could be explained in two different ways. First, strengthening may occur only for more recent memories. Second, older memories may not be reactivated [6], [12]. To decide which explanation is best to interpret our results, we performed the following experiments.

#### Older memories are subject to change by the compromised effect of interference via forgetting

To evaluate whether older memories can be labilized and consequently interfered with, we performed an eight-day experiment using three groups (Figure 3B.1). For two groups, subjects learned a list of paired syllables on Day 1 (List 1). On Day 7, one of these groups received a treatment. The one cue-reminder List 2 group (RcX1-L2) received one cue reminder, Rc, after which the List 2 learning task was completed. The no reminder group (NR 8d) did not receive any treatment on Day 7. The control List 2 group (CT-L2) learned List 2 on Day 7. All subjects were tested on Day 8.

First we analyzed List 2 performance at training and testing sessions. A repeated-measures ANOVA revealed no significant differences between the groups at List 2 training (Figure S3.F, F(1,18) = 1,609, p = 0,220) as well as no group by trial interaction (F(8,144) = 1,078, p = 0,381). An analysis of the percentage of correct responses given during the last four training trials yielded no significant difference between the groups at training (Figure S3.F inset, F(1,18) = 1,967, p = 0,177).

The List 2 performances on Day 8 for each group were estimated using the mean of total errors made in response to the List 2 cue syllables given in the in two testing trials. Subjects who received one cue-reminder on Day 7 followed by List 2 learning task (RcX1-L2) behaved in a way similar to subjects who learned List 2 on Day 7 only (CT-L2; Figure 3B.2.). Thus, for the List 2, the RcX1-L2 and L2 groups made a similar number of errors (F(1,18) = 2,138, p = 0,160). This result revealed the absence of RIF.

Then when we analyzed List 1 performance, a repeated-measures ANOVA revealed no significant differences between the groups List 1 training (Figure S4.G, F(1,20) = 1,861, p = 0,187) as well as no group by trial interaction (F(8,160) = 0,984, p = 0,450). An analysis of the percentage of correct responses given during the last four training trials yielded no significant difference between the groups at training (Figure S4.G inset, F(1,18) = 0,970, p = 0,759).

Next, we analyzed List 1 performance at testing. The number of errors made for List 1 were similar in both groups (Figure 3B.3 NR 8d and RcX1-L2 F(1,18) = 1,791, p = 0,204). The analysis of the type of errors made revealed no significant differences between the groups for void-type and intra-list errors; however, significant differences for confusion-type errors were observed (Figure 3B.4.2, p = 0,911, p = 0,187 and p = 0,034, respectively). Despite the absence of significant differences, confusion-type errors may indicate that there was an effect of the treatment, although, this effect may not have been large enough to be reflected in the total number of errors.

Considering the lack of significant differences in List 1 performance and the high number of errors made by the NR 8d group, we proposed that the absence of RIF could reflect not only interference but also forgetting of List 1 over time.

To confirm the interpretation exposed above, we compared the performance of two groups that learned List 1 on Day 1, one group was evaluated on Day 3, and the other group was evaluated on Day 8 (Text S2 and Figure S2). A greater number of total errors were reveled for the group evaluated on Day 8, exposing the effect of forgetting. Therefore, the presence of forgetting appears to overshadow the effect of interference on target memory (Figure 3B.3). And in this case, the absence of RIF on List 2 also revealed the effect of forgetting In addition, forgetting appears to impair memory enhancement as a consequence of repeated labilization-reconsolidations (Figure 3A.2).

## Discussion

The central conclusion of this paper states that the strengthening function of the reconsolidation process transforms the destiny of new memories. This conclusion emerges from the analysis in two different scenarios. First, strengthening of the original memory by repeated labilization-reconsolidations maintained the memory available for longer periods of time. In addition, we demonstrated not only that two successive reactivations improved the persistence of the memory but also that just one reactivation induced better performance at testing long after acquisition. This effect does not depend solely on retrieval; simple recall does not make the memory available for longer periods of time. In previous reports, we have demonstrated that mere retrieval does not affect the stability of retrieved memories, which is invulnerable to different treatments [20], [21]. Memories can be reactivated, and, consequently, they are potentially susceptible to strengthening. Second, when a memory is labilized by the presentation of the proper reminder and the process is retriggered after another cue reminder is presented in the time window of the first, subjects’ performance can improve upon testing on Day 3. Reinforced memories are more resistant to interference; being the effect of the second task less impairing to re-stabilization of the original memory.

As in previous studies, we analyzed the types of error observed during testing. Two different patterns of error reduction were found depending on the time of testing. Improvement in performance on Day 7 is expressed via reduced void-type errors (when the subjects did not answer). Thus, the improvement in memory persistence is reflected by remembering the whole response syllable. On the other hand, and as we have demonstrated before [15], the groups that received two cue reminders on Day 2 made fewer confusion-type errors (either one wrong letter in a group of three or three correct letters but in the wrong order) than the other groups that were evaluated on Day 3. This supports the idea that here the improvement is shown by increased memory precision.

More interestingly, when we analyzed the performance of the group that received two memory reactivations followed by an interfering task (RcX2-L2) an amnesic effect was revealed (Figure 2A.3 RcX2-L2 vs. RcX2). However, a comparison between the performance of this group and the group that received one memory reactivation revealed that performance was similar (Figure 2A.3; RcX2-L2 vs. RcX1). Thus, it appears that memory passes through both the strengthening and impairment processes during re-stabilization. It is also possible that interference selectively cancelled the strengthened effect of the second reactivation. It is clear that the strengthened memory is more resistant to the onslaught of amnesic agents, even if the mechanism by which this occurs is not fully understood. In line with these results, Wichert and co-workers [24] used the picture recognition paradigm to demonstrate that memory is still sensitive to interference from a second learning task despite repeated reactivations. Moreover, using contextual fear conditioning, it has been shown that repeated reactivations reduce the threat of hippocampal damage [25].

Even more interesting was the difference between performances after one labilization-reconsolidation when tested 24 h or 120 h after the reminder presentation. One reactivation alone was enough to make the memory more accessible for longer periods of time. Thus, the results obtained for one cue-reminder evaluated with different intersession intervals seems to contradict each other. However, it might be speculated that the absence of an effect when the memory is evaluated on Day 3 is the result of a ceiling effect. The passage of time associated with forgetting (Text S2 and Figure S2) is the essential factor to reveal such an effect and this occurs at a long term test on Day 7 (when the subjects committed a high number of errors).In other words, the strengthening effect of one cue-reminder is revealed on Day 7 but not on Day 3. On Day 7 there is a considerable forgetting, however if a cue-reminder is presented on Day2 augments the persistence of the memory leading a low number of errors on Day7. On Day 3, when the forgetting is absent the strengthening effect of one cue-reminder could not be reveal.This report shows this effect of strengthening after only one labilization of the target memory [7], [8], [16], [26] without any other treatment [27], [28]. Indeed, this result with one reactivation fits accurately with the initial description of the term reconsolidation [8].

Another contribution of this report is that strengthening is not active in older memories. When we presented one, two, or four cue reminders 7 days after training the memory was not reinforced by the treatment (Experiment 3A, Figure 3A). Indeed, subjects obtained a similar number of total errors as did a group without memory reactivation. We analyzed two working hypothesis for this. First, the memory may not be reactivated 7 days after training; second, although the memory can be reactivated, repeated labilizations cannot reinforce it. We tested the first hypothesis by reactivating the memory on Day 7 and immediately presented the second learning task as an interfering agent (Experiment 3B, Figure 3B). The results showed that there were no significant differences in the performance in terms of the total number of errors made when the target memory and the interfering task were evaluated. However, the confusion-type error rates were different between the control and interference groups (RcX1 and RcX1-L2, respectively), which might reflect that the target memory is in some way affected (Experiment 3B Figure 3B.4). Under these experimental conditions, another factor may be at play. Thus, the simple passage of time implies a forgetting process, which, in turn, changes the performance observed in all of the groups. Therefore, forgetting may overshadow the effect of interference. One way we probed this was by comparing subjects who acquired the memory at the same time point and were tested 3 or 8 days after training. This comparison showed that the effect of forgetting was reflected in both the total number of errors and number of void-type errors (Text S2 Figure S2). Thus, when the memory was reactivated 7 days after training, forgetting modified the effect of the interfering agent.

Hence, in the present case, the absence of effect does not depend on the absence of reactivation. Rather, it depends on the possibility of strengthening via repeated reactivations. Supporting this outcome, and using this paradigm Coccoz and colleagues [27] have shown that is possible to reactivate the declarative memory and to improve it when it was reactivated 6 days after training and a mild stressor (Cold Pressor Stress, CPS) was applied.

It has been suggested that the functional role of reconsolidation, whether induced by a second training trial or a non-reinforced reminder [12], [15], [16], is to mediate memory strengthening and prevent forgetting [12]. Here we have demonstrated that for human declarative memories, and this functional role of reconsolidation is constrained by the age of the memory. Reconsolidation may occur only in new memories, as demonstrated in animal models [12]. Moreover, Wichert et. al [29] showed that a 7-day-old memory was susceptible to interference by a second learning task, but a 28-day-old memory was not.

Using cue or contextual fear conditioning, different reports have shown that strong memories are more resistant to reactivation and interference of different amnesic agents [6], [18], [30]. Here, a different situation may occur. Indeed, younger and older memories can be reactivated, but only younger memories can be strengthened by reconsolidation. Hence, it could be considered that opposite effects may be observed when different factors are combined, such as the emotional charge of the memory, the age of the memory at the time of reactivation, and the parametrical conditions of the reminder. Supporting the idea that different outcomes could be obtained when memories are emotional or neutral, Schwabe and Wolf [31] examined the effect of stress on the reconsolidation of autobiographical memories in healthy human beings. Stress applied after memory reactivation impaired the memory of neutral episodes one week after recall, whereas the memory of emotional episodes was not affected.

The standard consolidation theory (SCT; [32], [33]) posits that the hippocampus is only a temporary storage area for memory and that the neocortex stores memories thereafter. However, some evidence seems to be incompatible with SCT. Consequently, Nadel & Moscovitch [34] proposed another theory, the multiple-trace theories (MTT), which hypothesizes that the hippocampal complex (HPC) rapidly encodes all episodic information. This information is sparsely encoded in distributed assemblages of HPC neurons, which act as an index of neocortical neurons that attend to the information and binds them into a coherent representation. The MTT also argued that reactivating a memory leads to the re-encoding and expansion of previously stabilized memory. Hence, this alteration of previously stored memories as a function of reactivation, provides a theoretical framework for understanding the reconsolidation effects [35].

Finally, the schema assimilation model (SAM, [36]) argues that newly acquired memories are not stored in isolation, but rather, new memories are gradually incorporated into a “schema,” or an organization of related knowledge. Using this framework the results reported here might imply that when the trace is neocortical (SCT), an ensemble of multiple traces (MTT), or the rapid incorporation of new information into the preexisting schema (SAM), the possibility of strengthening via repeated triggering of the reconsolidation process varies over time. Thus, it is possible speculate that for new memories, one reactivation is mounted on the previous reactivation; the trace might be reinforced by repeated activation of the same molecular pathway. However, when older memories include multiple traces in different brain regions, makes the possibility of reinforcing the single original trace less effective.

Understanding the dynamics of the reconsolidation of a neutral declarative memory and the inter-relatedness of these processes and the age and strength of the memory, are crucial to developing treatments for disorders and diseases with episodic, verbal, and prospective memory impairments in psychiatric (i.e., schizophrenia, bipolar disorder, autism) and neurological disorders (Alzheimer disease, temporal lobe epilepsy, stroke, brain injury, etc.) [37], [38]. Strengthening could be a novel behavioral strategy for cognitive rehabilitation. These treatments could enable patients to improve their memory precision and memory persistence in everyday life and are thus greatly needed.

## Materials and Methods

### Subjects

Two hundred and sixty undergraduate and graduate students from Buenos Aires University volunteered for the study. Only the subjects that achieved at least 60% of correct responses during the last four trials of the training session (12/20 correct responses) were included. Additionally, subjects were excluded for any of the following reasons: those who drank alcohol during the period of the experiment, those who wrote the syllables down, those who slept during the daytime after the reminder, and/or those who missed some step in the protocol of the experiment. Their ages ranged from 18 to 35, with a mean of 25. Each participant was randomly assigned to one of seventeen groups.

The protocol was approved by the Comité de Ética de la Sociedad Argentina de Investigación Clínica Review Board. Before their participation in the experiment, subjects provided written informed consent that had been approved by the Comité de Ética de la Sociedad Argentina de Investigación Clínica Review Board.

### Procedure

Experiments took place in a dark room and were conducted using a personal computer. Each subject was provided with earphones and seated facing a monitor placed in front of a large screen on the back wall.

The subjects were required to learn a list of five pairs of nonsense syllables presented on the monitor screen. In the first trial the List was shown and in the successive trials the five cue-syllables were presented and subjects had to write down the corresponding response-syllable. The List was associated with a specific context (light projected on a large screen, an image on the monitor screen; and a sound coming through the earphones).

**Figure 4:**
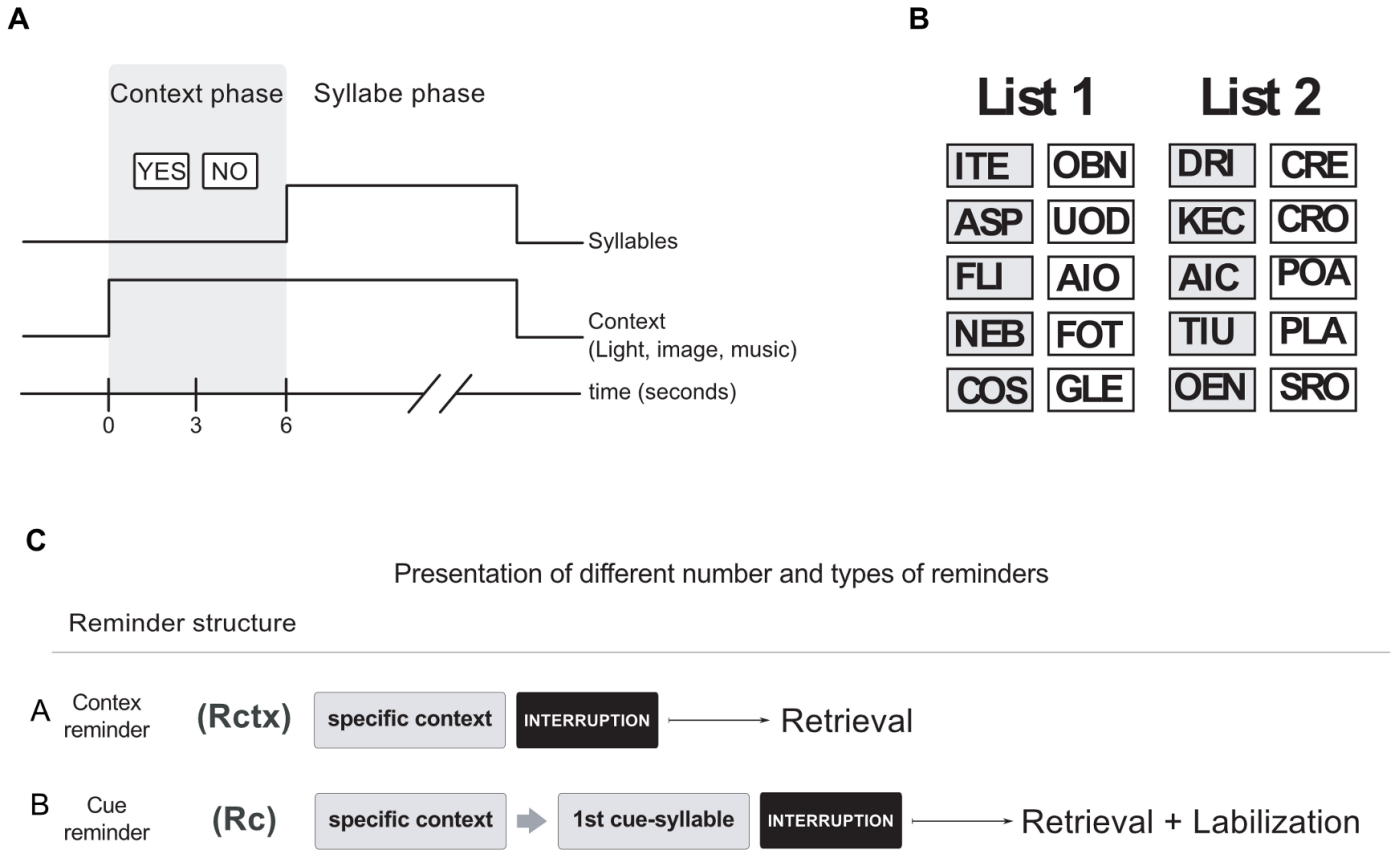
Experimental Protocol. **A) Actual trial.** It was formed by the context period: specific combination of a light (color illumination of the room), image (a picture on the monitor) and sound (music melody from earphones); and by a syllable period: six seconds after the stimuli presentation, five pairs of cue-response syllables were presented successively and in random order. **B) Paired-associated memory.** The List1 and List2 list presented in the training and testing sessions. **C) Types of reminders.** (*Top diagram*) **The cue reminder (Rc)** included the specific context, subjects had to press the expectancy keys (YES-NO), then one cue-syllable was presented after which the trial was abruptly interrupted, thus not allowing the subject to answer with the respective response-syllable. (*Middle diagram*) **The context reminder (Rctx)** consisted of the presentation of specific context, subjects had to press the expectancy keys (YES-NO) and the trial was abruptly interrupted before any syllable presentation. (*Bottom diagram*). Scissors stand for the full-stop of each type of reminder.

There were two types of trials, actual trials (specific context+List) and fake trials (contexts that were never followed by the List presentation). Each trial began with the 6-second presentation of the context period (Figure 4.A) but only actual trials were followed by the syllable presentation and the specific context, which persisted throughout (Figure 4.B).

#### The training session

Each trial was composed of the **context period** with diverse stimuli options: for the List 1 the light could be blue or green; the image, three different pictures of cascades; the sound, three different tango melodies. Only one combination of these options (the specific context) was followed by the syllables presentation of List 1 (syllable period). The context period for the List 2 there was two possible options: the light could be green or yellow; the image, a picture of a forests; the sound, three symphonies melodies. Only one combination of these options (the specific context) was followed by the syllables presentation of List 2 (syllable period).

The trial which includes the specific context followed by the syllables presentation is termed the actual trial while the others with only context (i.e., without syllables presentation) are called the fake trials.

The *syllable period* started with the presentation of a cue syllable on the left-hand side of the monitor screen and an empty response-box on the right. Each cue-syllable was taken at random from a list of five pairs. Subjects were given 5 s to write the corresponding response-syllable. Once that period was finished three situations were possible: first, if no syllable was written, the correct one was shown for 4 s; second, if an incorrect syllable was written, it was replaced by the correct one and it was shown for 4 s; and third, if the correct response was given, it stayed for 4 s longer. Immediately after that, another cue-syllable was shown and the process was repeated until the list was over. Altogether an actual trial lasted 51 s (6s for context period and 45 s for syllable presentation). Throughout this paper, every time a subject faced a cue-syllable and wrote down an erroneous response or no response an error was computed.

The training consisted of the presentation of 10 actual trials mixed with 22 fake trials (total: 32 trials), separated by a 4-s intertrial interval. In the first training trial the List was shown, and in the successive actual trials subjects were required to write down the corresponding response-syllable for each cue-syllable presented. The List 1 was composed of five pairs of nonsense cue-response syllables in rioplatense Spanish: **ITE-**OBN, **ASP**-UOD, **FLI**-AIO, **NEB**-FOT, **COS**-GLE (bold type: cue-syllable; regular type: response-syllable). The List 2 had the same proprieties: **OEN-**SRO, **DRI-**CRE, **AIC-**POA, **TIU-**PLA, **KEC-**CLO (Figure 4.B).

Fake trials were presented in order to enhance the level of attention and subjects were instructed to press the YES or NO button (the expectancy keys) on the keyboard 3 sec after the light–image–sound sequence had started (YES if they considered that it was the context associated to the List, NO in the opposite case). Therefore, this design allowed subjects to predict the presentation of the pair-associated task every time the specific context was completed. The training session lasted 15 min.

#### Testing session

The testing session consisted of 2 actual trials mixed with 5 fake trials (total: 7 trials each). The testing session lasted 2,5 min.

An error was computed every time a subject faced a cue-syllable and wrote down an erroneous response or no response.

During testing we were allowed to record what subjects write down. Thus, to perform a more deeply analysis the errors executed at testing were classified in three categories: Void-Type error, when no response was written down; Intralist-Type error, when the response-syllable was not the right one but it belonged to the List; Confusion-Type error, when the response-syllable was not included in the List.

#### Types of reminders

##### Context reminder (Rctx)

This trial included the context of the list (light,image,sound), subjects had to press the YES or NO button (the expectancy key) and immediately after the context period, before any syllable presentation, a notice displayed on the monitor announced that the session had to be suspended (Figure 4.C.A). It was demonstrated that this type of reminder does not trigger memory labilization reconsolidation.

##### Cue reminder (RcX)

This trial included the context of the list (light,image,sound), subjects had to press the YES or NO button (the expectancy key) and immediately after the context period, as expected, a cue syllable appeared on the left-hand side of the monitor screen and the response-box on the right. However, 2 s later a notice displayed on the monitor announced that the session had to be suspended, thus not allowing the subject to write down the response-syllable (Figure 4.C.B).

#### Demo

Before the training session, participants were confronted with a demo program to receive all the instructions and to understand the goal of the task. The program consisted of 4 trials, similar in structure to those of the training session, but with other contexts and two different pairs of nonsense-syllables.

### Experimental Groups

#### Experiment 1

*(n = 12).* Group NR 7d (no-reminder): Subjects received the training session (List 1) on Day 1 and were tested on Day 7. Group RcX1: Subjects received the training session (List 1) on Day 1, the cue reminder on Day 2 and were tested on Day 7. Group RcX2: Subjects received the training session (List 1) on Day 1, two cue reminder on Day 2 and were tested on Day 7.

#### Experiment 2

*(n = 13).* Group NR 3d (no-reminder): Subjects received the training session (List 1) on Day 1 and were tested on Day 3. Group RcX1: Subjects received the training session (List 1) on Day 1, the cue reminder on Day 2 and were tested on Day 3. Group RcX1– L2: Subjects received the training session (List 1) on Day 1, the cue reminder on Day 2 and five minutes later learned the List 2 and were tested on Day 3. Group Rcx2: The protocol was the same as Group RcX1 but they received the cue-reminder two times separated. Group Rcx2– L2: The protocol was the same as Group RcX2 but they learned List 2 five minutes later. Group CT – L2: Subjects learned List 2 on Day 2 and were tested on Day 3.

#### Experiment 3A

*(n = 10).* Group NR 8d: As in experiments 1A and 2 but were tested on Day 8. Group RcX1: Group RcX1: Subjects received the training session (List 1) on Day 1, the cue reminder on Day 7 and were tested on Day 8. Group RcX2: The protocol was the same as Group RcX1 but they received the cue-reminder two times separated. Group RcX4: As RcX4 but they received four cue-reminder.

#### Experiment 3B

*(n = 12).* Group NR 3d: As in Experiment 1A. Group NR 8d: As in Experiment 3.

#### Experiment S1

*(n = 12).* Group RcX1: Subjects received the training session (List 1) on Day 1, the cue reminder on Day 2 and were tested on Day 7. Group Rctx: Subjects received the training session (List 1) on Day 1, the context reminder (no- labilization) on Day 2 and were tested on Day 7.

#### Experiment S2

*(n = 10).* Group NR 8d: As in experiment 3. Group RcX1– L2: Subjects received the training session (List 1) on Day 1, the cue reminder on Day 7 and five minutes later learned the List 2 and were tested on Day 8. Group CT – L2: As in experiment 2.

### Statistics

#### Training Session

Mean number of errors per training-trial was reported and training curves were analyzed with repeated measures ANOVA.

#### Testing Session

Results were reported as mean number of total errors (block of first and second trial). Data from each experiment were first analyzed with one-way analysis of variance (ANOVA). It was followed by Post-hoc comparisons (FISHER, α = 0.05).

#### Types of errors

Void, Intralist and Confusion-Types were reported as mean number of errors (block of first and second trial) and were analyzed with one-way analysis of variance (ANOVA). It was followed by LSD Post-hoc comparisons (FISHER, α = 0.05).

## Supporting Information

Figure S1 (n = 12). B) The retrieval does not modify the memory persistence. **A.1) Experimental protocol.** A three-day experiment. Symbols as in experiment 1.A. Group RcX1 received a cue reminder on Day 2 and Group Rctx received a context-reminder. **A.2) Testing session.** Mean number of total errors +/− SEM on Day 7. Light gray bar stands for Group RcX1 and double stripe bar stands for Group Rctx.**A.3)** Error type. **A.3.1)**Mean number of Void-type errors +/− SEM on Day 7. **A.3.2)** Confusion-type errors. **A.3.3)** Intralist-type errors. Symbols as above.
(TIF)

Figure S2 Increasing the acquisition – testing interval reveals the forgetting on Day 8 Experiment S2 A (n = 10) **A.1) Experimental protocol.** A two-day experiment. Symbols as above. **A.2) L1 Testing Session.** Mean number of total errors +/− SEM on Day 3 and 8, Black bar stands for Group NR 3d and White bar for Group NR 8d. **A.3) Error Type. A.3.1)** Mean number of Void-Type error errors +/− SEM *,p<0,05. on Day 3 and 8. **A.3.2)** Confusion-type errors. **A.3.3)** Intralist-type errors. Symbols as above.
(TIF)

Figure S3 Learning curves. **Mean** number of errors +/−SEM per trial on Day 1. On the first trial the List is presented for the first time. **A)** **Experiment 1A.** Black rombhus stand for the Group NR 7d, White squares stand for the Group RcX1, white triangle for the Group RcX2. *Inset.* Mean number of total errors in the four last actual trials. Black bar stands for Group NR 7d, Gray bar for Group R_c_x1 and White bar for the Group RcX2. **B)** **Experiment S1**. Gray rombhus stand for the Group RcX1, White squares stand for the Group Rctx. *Inset.* Mean number of total errors in the four last actual trials. Gray bar stands for Group RcX1 and double stripe bar stands for Group Rctx. **C)** **Experiment 2.A.** ***List 2*** ** ***Training.*** Black rombhus stand for the Group CT-L2, grey squares stand for the Group RcX1-L2 and White rombhus stands for the Group RcX2-L2 *Inset*. Mean number of total errors in the four last actual trials. Black bar stands for Group CT-L2, grey bar for the Group RcX1-L2 and stripe bar for RcX2-L2. **D)** **Experiment 2.A.** ***List 1*** ** ***Training***. Light gray squares stand for the Group RcX1, Dark grey squares stand for the Group RcX1-L2, White triangles for the Group RcX2 and White rombhus for the Group RcX2-L2 *Inset*. Mean number of total errors in the four last actual trials. Light gray bar stands for the Group RcX1, Dark gray for RcX1-L2, white for the Group RcX2 and stripe for the Group RcX2-L2.**E)** **Experiment 3.A.** Black rombhus stand for the Group NR 8d, grey squares stand for the Group RcX1, White triangules for the Group RcX2 and White dots for the Group RcX4. L2 *Inset*. Mean number of total errors in the four last actual trials. Black bar stands the Group NR 8d, gray bar for the Group RcX1, White bar for the Group RcX2 and stripe bar for the Group RcX4. **F) Experiment 3.B.** ***List 2 Training.*** Black rombhus stands for the Group CT-L2 and gray squares for the Group RcX1-L2. *Inset*. Mean number of total errors in the four last actual trials. Black bar stands the Group CT-L2 and gray bar for the Group RcX1-L2.
(TIF)

Figure S4 Learning curves. Mean number of errors +/−SEM per trial on Day 1. On the first trial the List is presented for the first time.**G) Experiment 3.B.** ***List 1 Training.*** Black rombhus stands for the Group NR 8d and gray squares for the Group RcX1-L2. *Inset*. Mean number of total errors in the four last actual trials. Black bar stands for Group NR 8d and gray bar for the Group RcX1-L2. **H) Experiment S2.** Black rombhus stands for the Group NR 3d and White squares for the Group NR 8d. *Inset*. Mean number of total errors in the four last actual trials. Black bar stands the Group NR 3d and White Squire for the Group NR 8d.
(TIF)

Text S1 Description of Experiment S1: Retrieval does not modify memory persistence.
(DOC)

Text S2 Description of Experiment S2: Increasing the acquisition – testing interval reveals the forgetting on Day 8.
(DOC)
