# Supplementary figures and images for: The Role and Dynamic of Strengthening in the Reconsolidation Process in a Human Declarative Memory: What Decides the Fate of Recent and Older Memories?
Source: PLoS One. 2013 Apr 26;8(4):e61688. doi: 10.1371/journal.pone.0061688 (PMC3637303; doi:10.1371/journal.pone.0061688)

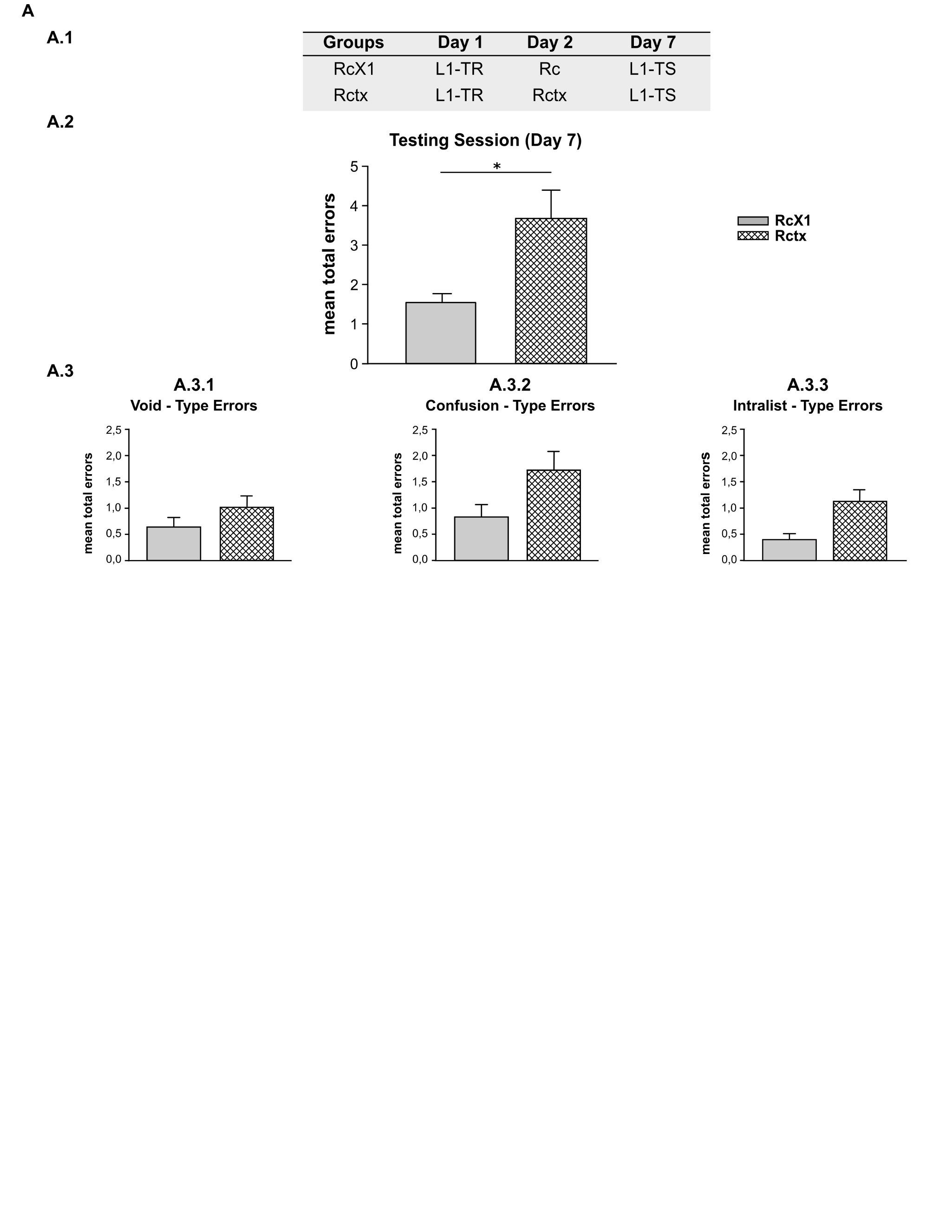

Supplement: Figure S1 — (n = 12). B) The retrieval does not modify the memory persistence. A.1) Experimental protocol. A three-day experiment. Symbols as in experiment 1.A. Group RcX1 received a cue reminder on Day 2 and Group Rctx received a context-reminder. A.2) Testing session. Mean number of total errors +/− SEM on Day 7. Light gray bar stands for Group RcX1 and double stripe bar stands for Group Rctx.A.3) Error type. A.3.1)Mean number of Void-type errors +/− SEM on Day 7. A.3.2) Confusion-type errors. A.3.3) Intralist-type errors. Symbols as above. (TIF) [file pone.0061688.s001.tif]

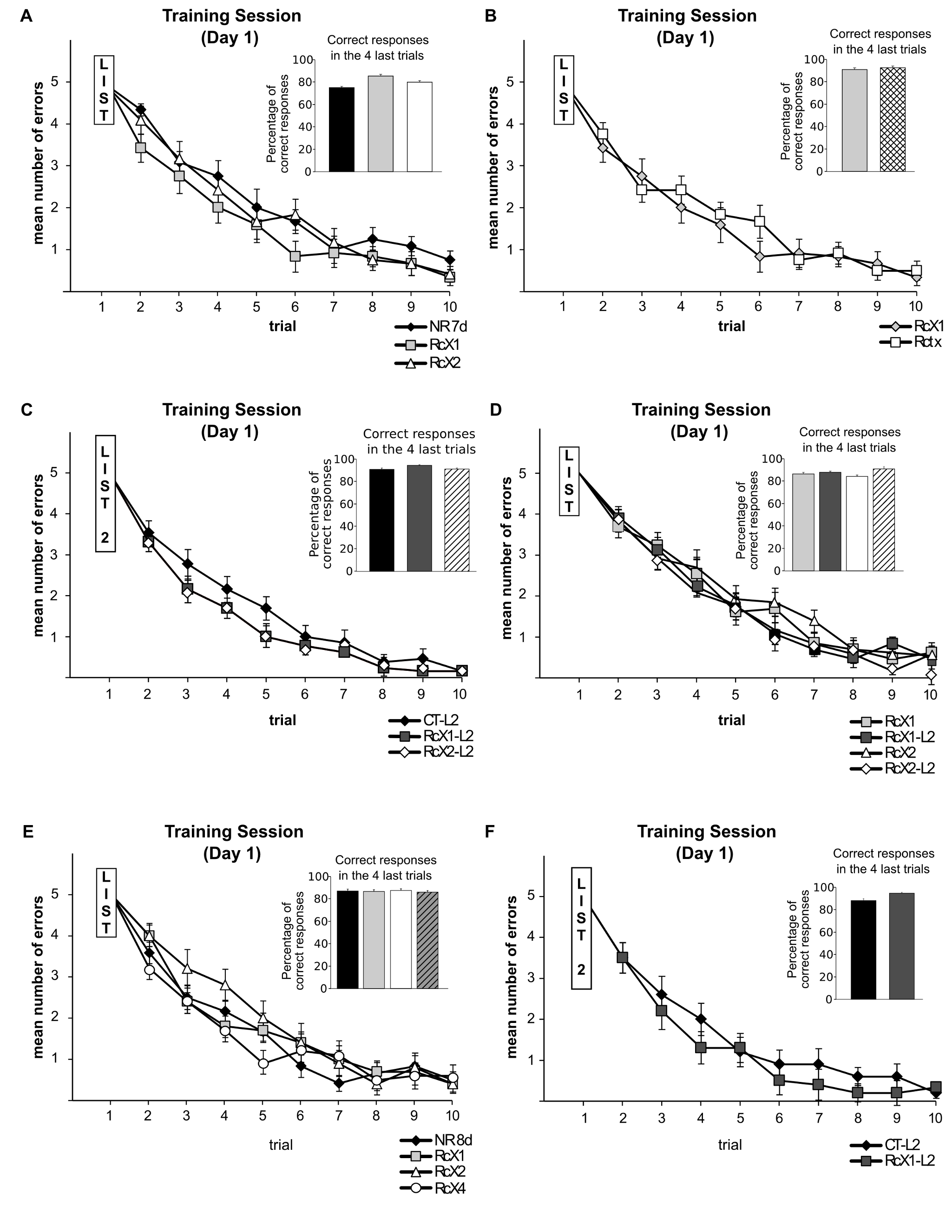

Supplement: Figure S3 — Learning curves. Mean number of errors +/−SEM per trial on Day 1. On the first trial the List is presented for the first time. A) Experiment 1A. Black rombhus stand for the Group NR 7d, White squares stand for the Group RcX1, white triangle for the Group RcX2. Inset. Mean number of total errors in the four last actual trials. Black bar stands for Group NR 7d, Gray bar for Group Rcx1 and White bar for the Group RcX2. B) Experiment S1. Gray rombhus stand for the Group RcX1, White squares stand for the Group Rctx. Inset. Mean number of total errors in the four last actual trials. Gray bar stands for Group RcX1 and double stripe bar stands for Group Rctx. C) Experiment 2.A. List 2 Training. Black rombhus stand for the Group CT-L2, grey squares stand for the Group RcX1-L2 and White rombhus stands for the Group RcX2-L2 Inset. Mean number of total errors in the four last actual trials. Black bar stands for Group CT-L2, grey bar for the Group RcX1-L2 and stripe bar for RcX2-L2. D) Experiment 2.A. List 1 Training. Light gray squares stand for the Group RcX1, Dark grey squares stand for the Group RcX1-L2, White triangles for the Group RcX2 and White rombhus for the Group RcX2-L2 Inset. Mean number of total errors in the four last actual trials. Light gray bar stands for the Group RcX1, Dark gray for RcX1-L2, white for the Group RcX2 and stripe for the Group RcX2-L2.E) Experiment 3.A. Black rombhus stand for the Group NR 8d, grey squares stand for the Group RcX1, White triangules for the Group RcX2 and White dots for the Group RcX4. L2 Inset. Mean number of total errors in the four last actual trials. Black bar stands the Group NR 8d, gray bar for the Group RcX1, White bar for the Group RcX2 and stripe bar for the Group RcX4. F) Experiment 3.B. List 2 Training. Black rombhus stands for the Group CT-L2 and gray squares for the Group RcX1-L2. Inset. Mean number of total errors in the four last actual trials. Black bar stands the Group CT-L2 and gray bar for the Group RcX1-L2. (T [file pone.0061688.s003.tif]

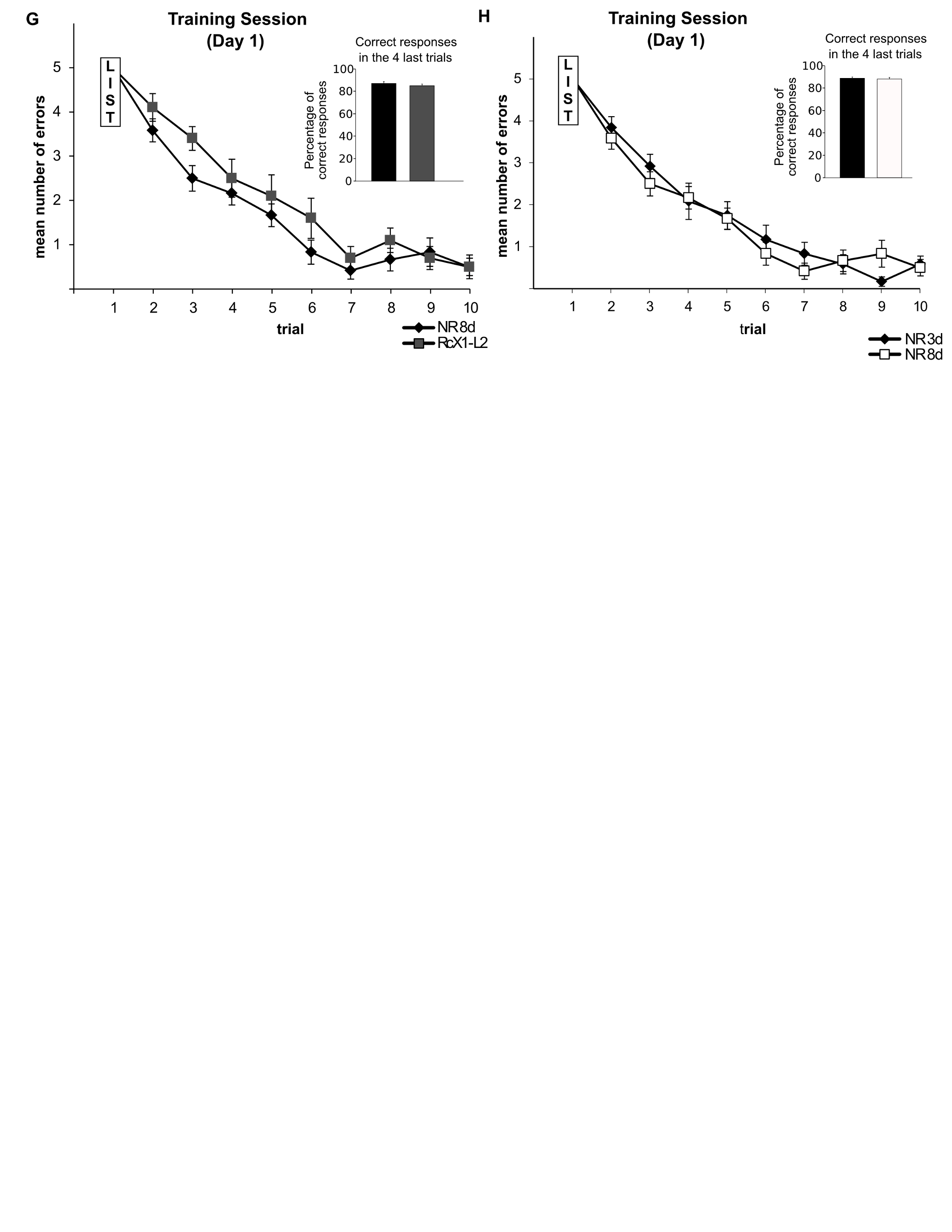

Supplement: Figure S4 — Learning curves. Mean number of errors +/−SEM per trial on Day 1. On the first trial the List is presented for the first time.G) Experiment 3.B. List 1 Training. Black rombhus stands for the Group NR 8d and gray squares for the Group RcX1-L2. Inset. Mean number of total errors in the four last actual trials. Black bar stands for Group NR 8d and gray bar for the Group RcX1-L2. H) Experiment S2. Black rombhus stands for the Group NR 3d and White squares for the Group NR 8d. Inset. Mean number of total errors in the four last actual trials. Black bar stands the Group NR 3d and White Squire for the Group NR 8d. (TIF) [file pone.0061688.s004.tif]
