# Supplementary material for: The Role and Dynamic of Strengthening in the Reconsolidation Process in a Human Declarative Memory: What Decides the Fate of Recent and Older Memories?
Source: PLoS One. 2013 Apr 26;8(4):e61688. doi: 10.1371/journal.pone.0061688 (PMC3637303; doi:10.1371/journal.pone.0061688)
Supplement: Text S1 — Description of Experiment S1: Retrieval does not modify memory persistence. (DOC) [file pone.0061688.s005.doc]

**SUPPORTING INFORMATION**

**Experiment S1. Retrieval does not modify memory persistence.**

In a previous report, we demonstrated that repeated destabilization of the original memory can strengthen it if subsequent destabilizations occur in the time window of the first re-stabilization. This effect depends on successively triggering reconsolidation, not from successive retrievals [15]. A main difference emerged when we evaluated the memory 7 days after training by triggering the labilization-reconsolidation process only once (i.e., RcX1). To refute the notion that this effect could be due simply to retrieval, we performed the following experiment. Our paradigm offers various reminders [20] to distinguish between these contrasting interpretations, namely, that memory retrieval, rather than memory reactivation, reinforces the original memory. Indeed, we have shown that omitting one aspect of a reminder­ retrieves the memory but deactivates the reminder. As a consequence, labilization of the target memory is prevented. Thus, to evaluate the effect of retrieval only on strengthening the target memory and consequently changing its persistence, we performed a seven-day experiment using two groups (Figure S1 A.1). On Day 1, subjects learned a list of paired syllables (List 1). On Day 2, they received a treatment. The one-cue reminder group received one cue reminder (RcX1), and the context-reminder group (Rctx) was exposed to one context reminder. The context reminder session was created using the context of the list (light,image,sound). Subjects had to press the YES or NO button (the expectancy key) and immediately after the context period, before any syllable presentation, a notice displayed on the monitor announced that the session had to be suspended (Figure 4.C.A). Finally the subjects had to leave the experimental room and were told that the experiment was over. The cue reminder session consisted in the context of the List 1 memory (light, image ,sound) as described above and the presentation of one cue syllable before been interrupted (Figure 4.C.B). Finally, all the subjects were tested on Day 7.

A context reminder does not enhance performance on Day 7.

We observed similar performance between the RcX1 group and the Rctx group during training (Figure S3.B, F(1,22)=0,364 p=0,551, interaction F(8,176) = 1,939 p = 0,056. Inset: F(1,22) = 0,082 p = 0,776). Subjects who received one cue reminder made fewer errors than subjects who were given one context reminder during the two testing trials on Day 7. Specifically, a significant difference between groups performance was observed during testing (Figure S1 A.2 F(1,22) = 10,565, p = 0,003). Moreover, a comparison of the types of errors made by each group revealed that equivalent numbers of each type of error were made by both groups Figure S1 A.3, Void-type: F(1,22) = 0,851 p = 0,366; Intra-list- type: F(1,22) =4,222 p = 0,051; Confusion-type: F(1,22) = 4,122 p = 0,054).

Therefore, retrieval on Day 2 did not increase memory persistence; this effect depends on the occurrence of at least the presentation of one cue-reminder to labilize the declarative memory.
