# Supplementary material for: The Role and Dynamic of Strengthening in the Reconsolidation Process in a Human Declarative Memory: What Decides the Fate of Recent and Older Memories?
Source: PLoS One. 2013 Apr 26;8(4):e61688. doi: 10.1371/journal.pone.0061688 (PMC3637303; doi:10.1371/journal.pone.0061688)
Supplement: Text S2 — Description of Experiment S2: Increasing the acquisition – testing interval reveals the forgetting on Day 8. (DOC) [file pone.0061688.s006.doc]

**SUPPORTING INFORMATION**

**Experiment S2. Increasing the acquisition – testing interval reveals the forgetting on Day 8.**

A repeated-measures ANOVA revealed no significant differences between the groups at training (Figure S4.H, F(1,22) = 0,097 p = 0,758) as well as no group by trial interaction (F(8,176) = 1,309, p = 0,241). An analysis of the percentage of correct responses given during the last four training trials yielded no significant difference between the groups at training (Figure S4.H inset, F(1,22) = 0,292, p = 0,865).

A greater number of total errors made by the NR 8d group exposed the effect of forgetting (Figure S2 A.2 One-way ANOVA F(1,22) = 5,727, p = 0,025, LSD post-hoc Comparison p=0,025). The same profile was obtained for void-type errors in this group (Figure S2 A.33.; F(1,22) = 5,353, p = 0,030). Thus, this type of error was considered to be a good predictor to evaluate the forgetting on older memories (i.e., 7 or 8 days after the training session). Therefore, this experimental condition demonstrated that forgetting could weaken the competence between both memories during the retrieval phase; this may be the principal factor determining the absence of RIF effect (Figure 3B.2).
